# Quinary Prevention in Patients With Type 2 Diabetes Mellitus Included in the Family Adoption Programme of a Medical College in Jaipur: A Qualitative Study

**DOI:** 10.7759/cureus.114089

**Published:** 2026-08-06

**Authors:** Sudeepti S Panat, Nitish Kumar, Purva Shoor, Vishal Bankwar

**Affiliations:** 1 Community Medicine, Shri Gorakshnath Medical College Hospital and Research Centre, Gorakhpur, IND; 2 Community Medicine, Jaipur National University Institute for Medical Sciences and Research Centre, Jaipur, IND; 3 Community Medicine, Gajra Raja Medical College, Gwalior, IND; 4 Community Medicine, Varun Arjun Medical College and Rohilkhand Hospital, Shahjahanpur, IND

**Keywords:** de-hearsay, focus group discussion, misinformation, quinary prevention, type-2 diabetes mellitus

## Abstract

Background

Diabetes-related misinformation adversely affects treatment adherence, health-seeking behaviour and disease outcomes among individuals with type 2 diabetes mellitus (T2DM). Quinary prevention aims to prevent harm arising from the spread of inaccurate health information by promoting evidence-based communication and countering misconceptions. However, evidence regarding diabetes-related misinformation and Quinary prevention strategies in community settings remains limited.

The study was mainly aimed at exploring misinformation related to T2DM management and its sources and exploring the Quinary prevention strategies employed by local practitioners and barriers faced by them in countering misinformation.

Materials and methods

A qualitative exploratory study was conducted in Khatipura and Luniyawas among diabetic patients using focus group discussions. A total of 31 patients were identified: 16 in Khatipura and 15 in Luniyawas, while five local practitioners were interviewed using a pilot-tested interview guide. Data included patients’ statements verbatim showing diabetes-related misconceptions, measures taken by the local practitioners and the barriers faced by them in countering such misinformation. Descriptive results were displayed using tables and pie charts.

Results

The most common beliefs included that allopathic medicines lead to multi-organ failure, insulin is the end stage in diabetes and twice-daily consumption of bitter gourd and aloe vera juice reverses diabetes. Counselling of patients was the most frequently adopted Quinary prevention strategy by the local practitioners. The most common barriers faced by local practitioners in countering misinformation included patient resistance to medical advice and over-reliance on quacks. Informal healthcare providers were the primary source of misinformation.

Conclusion

Diabetes-related misinformation remains highly prevalent and poses a significant challenge to effective T2DM management. The efforts of local practitioners in tackling the misinformation involved Quinary prevention, which was constrained by several factors such as poor health literacy, patient resistance to medical advice, over-reliance on quacks and long-standing faith in home remedies.

## Introduction

The incidence and prevalence rates of type 2 diabetes mellitus (T2DM) have quadrupled in the last 40 years globally and are still rising [1]. The International Diabetes Federation has reported that the global prevalence of T2DM is 9.3% in the age group of 20-79 years, accounting for 463 million cases worldwide [2]. A vast majority of factors are responsible for the increase in T2DM cases, such as obesity, smoking, tobacco consumption, alcohol intake, sedentary lifestyle, family history of T2DM, ageing, higher socioeconomic status and urbanisation [3]. T2DM is a complex metabolic disease that occurs as a result of complex interactions between genetic and environmental factors [4]. Therefore, it is necessary to implement strategies for the effective management of T2DM. Misinformation or reliance on de-hearsay is an important factor that presents a challenge to local practitioners, as it can affect the health-seeking behaviour of patients and outcomes of diabetes treatment. The technological advances and internet accessibility in remote areas have led to rapid dissemination of information among the masses, leading to self-management of diabetes and increasing the burden on local practitioners in dealing with its impacts [5]. Preventing the spread of misinformation regarding the management of T2DM has gained utmost importance in today’s digital world. To deal with the issue of the spread of misinformation, a fifth step was added to the traditional model comprising four steps of prevention known as Quinary prevention [6].

The word quinary is derived from a Latin word “quinarus,” which means five and describes the fifth object in a sequence of five. Quinary prevention aims at minimising the spread of incorrect health information and its impact on disease progression [7-9].

Quinary prevention is defined as “a set of policies and actions that hinder the spread of misinformation regarding health maintenance, disease prevention and management that increase the risk of spread of misinformation related to maintenance of health, prevention of disease and its management, thereby reducing its impact on disease progression” [9].

In simpler terms, it is defined as “preventing the dissemination of health-related hearsay or misinformation and its adverse effects on the health of the masses” [10].

T2DM is a significant public health problem both in India and globally, with prevalence rising due to rapid urbanisation, changing lifestyles and an increasing elderly population. The International Diabetes Federation has reported that India is one of the major contributors to the global diabetes burden, highlighting the need for effective diabetes management and patient education strategies [2].

Despite considerable progress in diagnostic and therapeutic approaches, misconceptions related to T2DM continue to persist, especially in low- and middle-income countries. Such misinformation directly impacts health-seeking behaviour, treatment adherence and the outcome of the disease [11].

A cross-sectional study conducted in North India by Rai and Kishore reported that a vast majority of individuals attributed the disease to excess sugar consumption, neglecting the role of genetic factors, insulin resistance and obesity [12].

Another prevalent misconception is that insulin therapy is addictive or signifies the terminal stage of the disease, leading to delayed initiation of insulin therapy. Furthermore, a substantial proportion of individuals believe that diabetes can be cured through home remedies such as bitter gourd or alternative therapies, thus resulting in discontinuation or modification of prescribed treatment [13,14].

It is commonly believed that diabetes does not occur in the absence of family history, leading to underestimation of personal risk and delayed screening [15]. Misconceptions related to monitoring of blood glucose levels have also been reported, reflecting inadequate diabetes-related knowledge [16].

The present study will be conducted in the peri-urban areas - Khatipura and Luniyawas (adopted by the medical college as a part of the Family Adoption Programme (FAP)) - to study the misinformation regarding the management of T2DM prevalent in that area and the sources responsible for dissemination of such information and the action taken by physicians practising in those areas to deal with this issue.

The study was conducted with the objectives of exploring the misinformation related to the management of T2DM prevalent among the study participants and identifying the various sources of such misinformation. In addition, the study sought to explore the Quinary prevention strategies employed by private practitioners of modern medicine to address diabetes-related misinformation and to identify the barriers they face in countering misinformation in their routine clinical practice.

## Materials and methods

A qualitative exploratory study (using focus group discussions (FGDs)) was carried out among diabetic patients residing in the Khatipura and Luniyawas areas, which are located in the peri-urban region of Jaipur, the capital city of Rajasthan state in the western part of India. A list of known cases of T2DM diabetics residing in Khatipura and Luniyawas was prepared with the help of MBBS students of Jaipur National University Institute of Medical Sciences located in Jaipur under the FAP. All persons suffering from T2DM and local practitioners practising in the study areas were our study population. The study was carried out after obtaining ethical approval (Ref. No: JNUIMSRC/IEC/2024/14/ dated 19/12/2024) from January 2025 to June 2025.

Selection criteria

The study included known cases of T2DM of more than one year's duration, aged 45 years and above, who were permanent residents of the study area residing there continuously for at least six months and were willing to provide written informed consent to participate in the study. Allopathic practitioners who were willing to participate were also included. Individuals suffering from any mental illness or who were critically ill at the time of data collection were excluded from the study. In addition, AYUSH (Ayurveda, Yoga and Naturopathy, Unani, Siddha, and Homoeopathy) practitioners were excluded from participation.

Sampling and data collection 

In FAP, each MBBS student adopts different families from villages, and a house-to-house survey is done to identify the health needs of the family and the corresponding community. During this survey, a total of 31 patients of T2DM were identified fulfilling our selection criteria: 16 in Khatipura and 15 in Luniyawas area. The quantitative data, such as height, weight and waist circumference, were recorded by MBBS students during their family visit under FAP. A semi-structured interview guide (Appendices) was developed in Hindi and validated through a pilot study for conducting FGDs on such patients. In both areas, a total of five modern allopathic practitioners were identified by MBBS students during FAP; excluding informal and AYUSH practitioners, two in Khatipura and three in Luniyawas were interviewed after obtaining their written informed consent for the study. Thematic saturation was achieved as no new themes and sub-themes emerged in the final FGD conducted for patients and in-depth interviews conducted for local practitioners of modern medicine, and the data became repetitive with respect to study objectives.

Procedure for Conducting FGDs

Four FGDs were held. Two each in Khatipura and Luniyawas. Three FGDs comprised eight participants each, while the fourth included seven participants, resulting in a total of 31 participants. The FGDs were organised to ensure representation from both study areas while maintaining a recommended group size of six to 10 participants to facilitate active participation and meaningful interaction. Each FGD lasted approximately 35-40 minutes and was conducted at a community hall.

A training session was conducted one week prior to the camp to familiarise the students with the objectives of the study and the procedure for carrying out FGDs. The FGD team comprised five members, including two investigators and three MBBS students. The first investigator served as the moderator and facilitated the FGD, while the second investigator was responsible for preparing the sociogram to document participant interactions. Of the three MBBS students, two were assigned to carry out audio-visual recording of the discussion and document important observations and field notes, whereas the remaining student assisted the second investigator in preparing the sociogram. This role allocation ensured systematic data collection and smooth conduct of the FGDs.

A pilot study was carried out to check the feasibility of the study. It also sensitised the team regarding the study procedures and to tackle difficulties during the data collection. Before the commencement of FGDs, sufficient time was taken to build rapport with the study participants; then the objectives of the study were explained to them, and written informed consent was obtained from them for participating in the study.

Data Collection From Study Participants

Consecutive sampling technique was used to collect data from the study participants. A pilot-tested interview guide was developed in the local language (Hindi) for ease of understanding by study participants. Data regarding the socio-demographic profile of study participants, health-seeking behaviour and their dependence on de-hearsay for management of T2DM were collected during the FGDs.

Data Collection From Local Practitioners

Individual in-depth interviews were conducted with all five practitioners of modern (allopathic) medicine practising in the area after explaining to them the purpose of the study and obtaining their written informed consent. The interview guide explored their experiences with treating diabetes patients, sources of misinformation encountered in clinical practice, Quinary prevention strategies employed to counter misinformation and perceived barriers to addressing misinformation among patients. Each interview lasted approximately 20-30 minutes.

Data analysis

Audio recordings of FGDs were transcribed verbatim. Qualitative data were analysed manually using thematic analysis. Two investigators independently reviewed the transcripts, generated initial codes, grouped similar codes into categories and developed overarching themes. The Coding discrepancies between investigators were resolved through discussion and agreement, and the coding framework was refined until consensus was achieved. Representative quotations were selected to illustrate each theme. Socio-demographic characteristics were summarised into descriptive statistics generated with jamovi software (version 2.3.28, Jonathon Love, Damian Dropmann, and Ravi Selker, Sydney, Australia).

## Results

Table 1 summarises the socio-demographic and clinical characteristics of participants in the two study sites. In Khatipura (n = 16), most participants (11, 68.75%) were aged 45-59 years, and Hindus and Muslims were equally represented (eight, 50% each). All participants were married, and most lived in joint families (10, 62.5%) with lower educational attainment (11, 68.75%). Three-fourths were employed, and nearly all (15, 93.75%) belonged to a higher socio-economic status. Most participants led sedentary lifestyles (12, 75%) and were non-vegetarian (13, 81.25%). Just over half (nine, 56.25%) had been diabetic for one to five years, and a similar proportion were teetotallers, while seven (43.75%) were current smokers. Using Southeast Asian BMI cut-offs, half the participants were obese, and another seven (43.75%) were overweight; abdominal obesity (waist circumference >90 cm) was seen in 14, i.e., 87.5%.

**Table 1:** Socio-demographic and clinical profile of study participants (N = 31) BMI was classified according to the Southeast Asian BMI cut-offs: normal (18.5-22.9 kg/m²), overweight (23.0-24.9 kg/m²), and obese (≥25.0 kg/m², combining obese I and obese II). Abdominal obesity was defined as a waist circumference >90 cm in males.

Variable	Category	Participants n (%) - Khatipura (n = 16)	Participants n (%)- Luniyawas (n = 15)
Age group (years)	45-59	11 (68.75)	09 (60)
	>60	05 (31.25)	06 (40)
Religion	Hindu	08 (50)	08 (53.33)
	Muslim	08 (50)	07 (46.66)
Marital status	Married	16 (100)	15 (100)
	Unmarried	0 (0)	0 (0)
	Divorced/separated	0 (0)	0 (0)
Type of family	Joint	10 (62.5)	09 (60)
	Nuclear	06 (37.5)	06 (40)
Education	Higher education (higher secondary and above)	05 (31.25)	06 (40)
	Lower education (up to high school)	11 (68.75)	09 (60)
Occupation	Employed	12 (75)	14 (93.33)
	Unemployed	04 (25)	01 (6.66)
Socio-economic class (according to modified Kuppuswamy scale - September 2025)	Middle class and above (classes I-III)	15 (93.75)	15 (100)
	Lower class (classes IV-V)	01 (6.25)	0 (0)
Type of worker	Sedentary worker	12 (75)	09 (60)
	Moderate worker	02 (12.5)	05 (33.33)
	Heavy worker	02 (12.5)	01 (6.66)
Duration of diabetes	1-5 years	09 (56.25)	09 (60)
	More than 5 years	07 (43.75)	06 (40)
Family history	Present	07 (43.75)	05 (33.33)
	Absent	05 (31.25)	10 (66.66)
	Do not know	04 (25)	0 (0)
Diet preferences	Vegetarian	03 (18.75)	03 (20)
	Non-vegetarian	13 (81.25)	12 (80)
Alcohol consumption	Teetotaller	09 (56.25)	08 (53.33)
	Less than 3 days per week	06 (37.5)	02 (13.33)
	More than 3 days per week	01 (6.25)	05 (33.33)
Smoking status	Non-smoker	06 (37.5)	05 (33.33)
	Current smoker	07 (43.75)	07 (46.66)
	Ex-smoker	03 (18.75)	03 (20)
BMI category (Southeast Asian cut-offs)	Normal	01 (6.25)	04 (26.66)
	Overweight	07 (43.75)	08 (53.33)
	Obese	08 (50)	03 (20)
Abdominal obesity (waist circumference) (males)	> 90 cm	14 (87.5)	12 (80)
	< 90 cm	02 (12.5)	03 (20)

In Luniyawas (n = 15), nine (60%) participants were aged 45-59 years, with slightly more Hindus (eight, 53.3%) than Muslims (seven, 46.7%). All were married, most belonged to joint families (nine, 60%) with lower education levels (nine, 60%), and nearly all (14, 93.3%) were employed. Every participant belonged to a middle or higher socio-economic class. Sedentary behaviour was common (nine, 60%), followed by moderate activity (five, 33.3%). Diabetes duration was one to five years in nine (60%) participants and over five years in six (40%), with a third reporting a family history of diabetes. Most were non-vegetarian (12, 80%), just over half were teetotallers (eight, 53.3%), and a third drank alcohol more than three days a week. Nearly half (seven, 46.7%) smoked, and just over half (eight, 53.3%) were overweight by Southeast Asian BMI criteria. Abdominal obesity was present in 12, i.e., 80% of participants.

Table 2 summarises the prevalent misinformation related to T2DM among participants from Khatipura (n = 16) and Luniyawas (n = 15). In Khatipura, the common misconceptions included diabetes occurring due to consumption of sweets/fruits (11, 68.75%), diabetes disappearing after stopping sugar intake (nine, 56.25%), diabetes being controllable without allopathic medicines (six, 37.5%), absence of diabetes without a family history (three, 18.75%), and repeated testing causing diabetes (one, 6.25%). All participants (16, 100%) believed that allopathic medicines cause multi-organ failure and that insulin represents the end stage of diabetes, while eight (50%) considered insulin harmful and addictive. Additionally, 15 (93.75%) believed kidney function tests and lipid profile assessment were unnecessary if blood sugar was normal, 12 (75%) believed home remedies could cure diabetes, and 11 (68.75%) considered bitter gourd, coriander and aloe vera juice as cures.

**Table 2:** Prevalent misinformation related to T2DM among study participants (N = 31)

Sr. no.	Misinformation statement	Participants endorsing belief n (%) - Khatipura (n = 16)	Participants endorsing belief n (%) - Luniyawas (n = 15)
1	Diabetes occurs due to consumption of sweets/fruits	11 (68.75)	8 (50)
2	Diabetes does not occur without family history	03 (18.75)	03 (20)
3	Repeated testing for diabetes is responsible for the occurrence of diabetes	01 (6.25)	01 (6.66)
4	Diabetes disappears completely if a person stops consuming sugar	09 (56.25)	08 (53.33)
5	Diabetes can be controlled without allopathic medicines	06 (37.5)	04 (26.66)
6	Allopathic medicines can cause multi-organ failure	16 (100)	11 (73.3)
7	Insulin is the end stage in diabetes	16 (100)	12 (80)
8	Insulin is harmful and addictive	08 (50)	06 (40)
9	There is no need to check KFT and lipid profile if blood sugar readings are normal	15 (93.75)	15 (100)
10	It is not safe for diabetics to perform physical exercise	0 (0)	0 (0)
11	Five-minute yoga is better than 50 minutes aerobic exercise	0 (0)	0 (0)
12	Home remedies can cure diabetes	12 (75)	03 (20)
13	Bitter gourd, coriander and aloe vera juice can cure diabetes	11 (68.75)	06 (40)

In Luniyawas, misconceptions included diabetes occurring due to sweets/fruits (eight, 53.33%), diabetes disappearing after stopping sugar (eight, 53.33%), diabetes being controllable without allopathic medicines (four, 26.67%), absence of diabetes without family history (three, 20%), repeated testing causing diabetes (one, 6.67%), allopathic medicines causing multi-organ failure (11, 73.3%), insulin being harmful and addictive (12, 80%), kidney function tests and lipid profile being unnecessary if blood sugar was normal (15, 100%), home remedies curing diabetes (three, 20%), and bitter gourd, coriander, and aloe vera juice curing diabetes (six, 40%). None of the participants in either area believed that physical activity was unsafe for people with diabetes or that five minutes of yoga was superior to 50 minutes of aerobic exercise.

Table 3 represents representative verbatim quotations from study participants that illustrate the major misconceptions related to T2DM management identified during the FGDs. The quotations highlight misconceptions regarding the causes of diabetes, the perceived harmful effects of anti-diabetic medications, fear and misconceptions surrounding insulin therapy and the belief that home remedies can cure diabetes. Participants commonly associated diabetes with excessive consumption of sweets, believed that anti-diabetic drugs damage vital organs, considered insulin to be addictive and the final stage of diabetes, and trusted home remedies such as bitter gourd, aloe vera and coriander juice as cures. These quotations provide evidence supporting the themes identified in the analysis and show how misinformation affects diabetes management practices among the study participants.

**Table 3:** Representative verbatim quotations illustrating misconceptions related to T2DM management

Theme	Sub-theme	Illustrative verbatim statement
Misinformation related to aetiology of diabetes	Diabetes is caused by eating sweets.	“Eating too many sweets caused diabetes.” “I thought if no one in my family is diabetic, then I will also remain free from diabetes.” “If I stop eating sugar completely, I will get rid of diabetes.”
Misinformation related to anti-diabetic drugs	Allopathic medicines damage organs.	“I stopped taking anti-diabetic drugs as they caused side effects, now I rely on diet control alone to reduce my blood sugar levels.” “I prefer home remedies as anti-diabetic drugs can damage the organs.”
Misinformation related to Insulin	Insulin is the end stage of diabetes. Insulin is addictive.	“My friend told me that insulin, once started, has to be continued lifelong.” “Insulin is dangerous and I will get addicted to it.”
Misinformation related to cure of diabetes	Home remedies cure diabetes.	“Home remedies do not cause harm and can completely cure diabetes.” “Twice daily intake of bitter gourd, aloe vera and coriander juice can reverse diabetes and can completely stop the need of medicines.”

**Figure 1:**
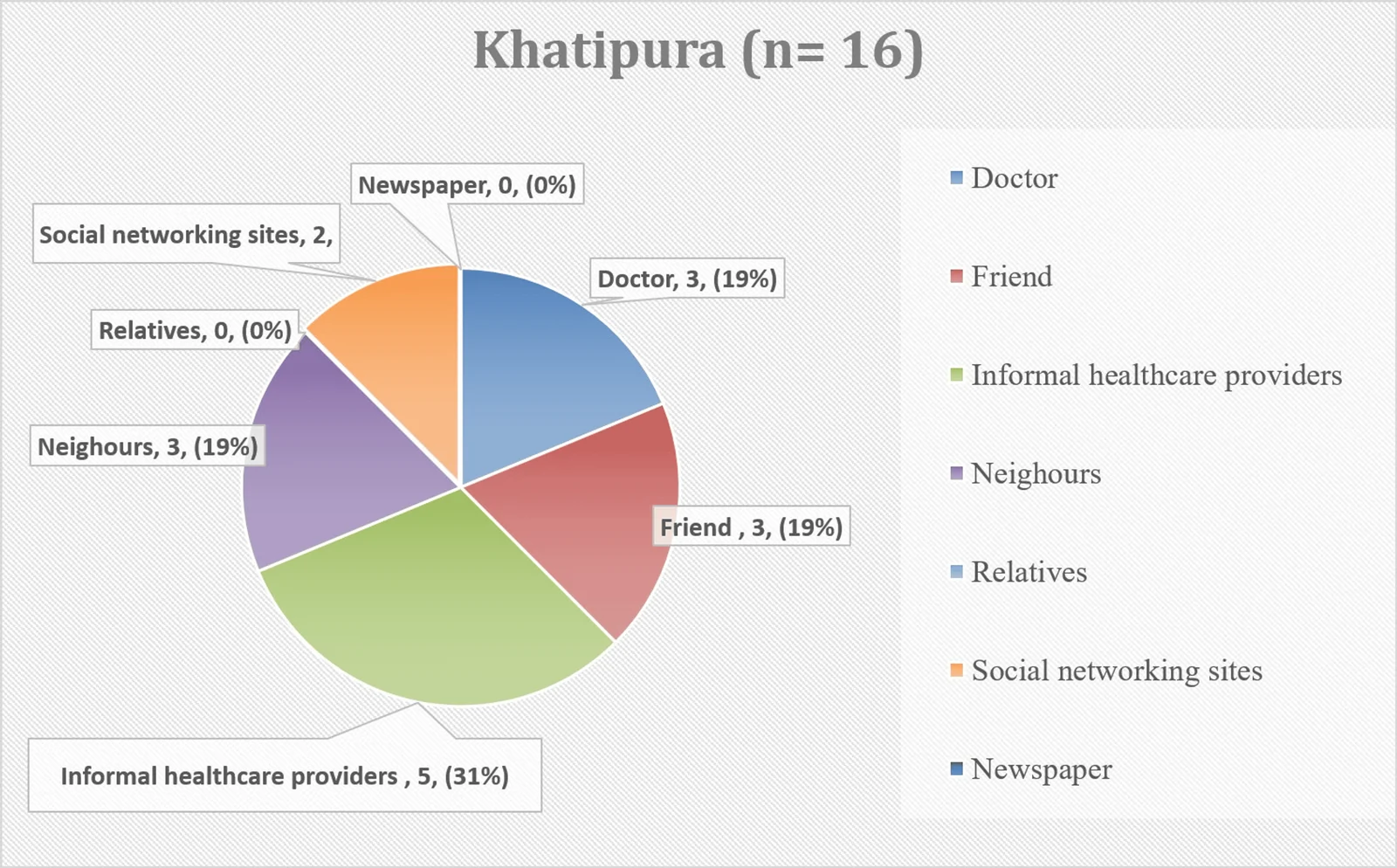
Distribution of study participants according to their sources of health information in Khatipura (n = 16) Figure created using Microsoft Word (Microsoft® Corp., Redmond, WA).

Figure 1 depicts the distribution of study participants according to their sources of health information in Khatipura (n = 16), where informal healthcare providers were the predominant source of health information reported by five participants (31.3%). This was followed by doctors, friends and neighbours, each reported by three participants (18.8%), while social networking sites accounted for 12.5%, reported by two participants. None of the participants reported obtaining health information from relatives or newspapers (0%).

**Figure 2:**
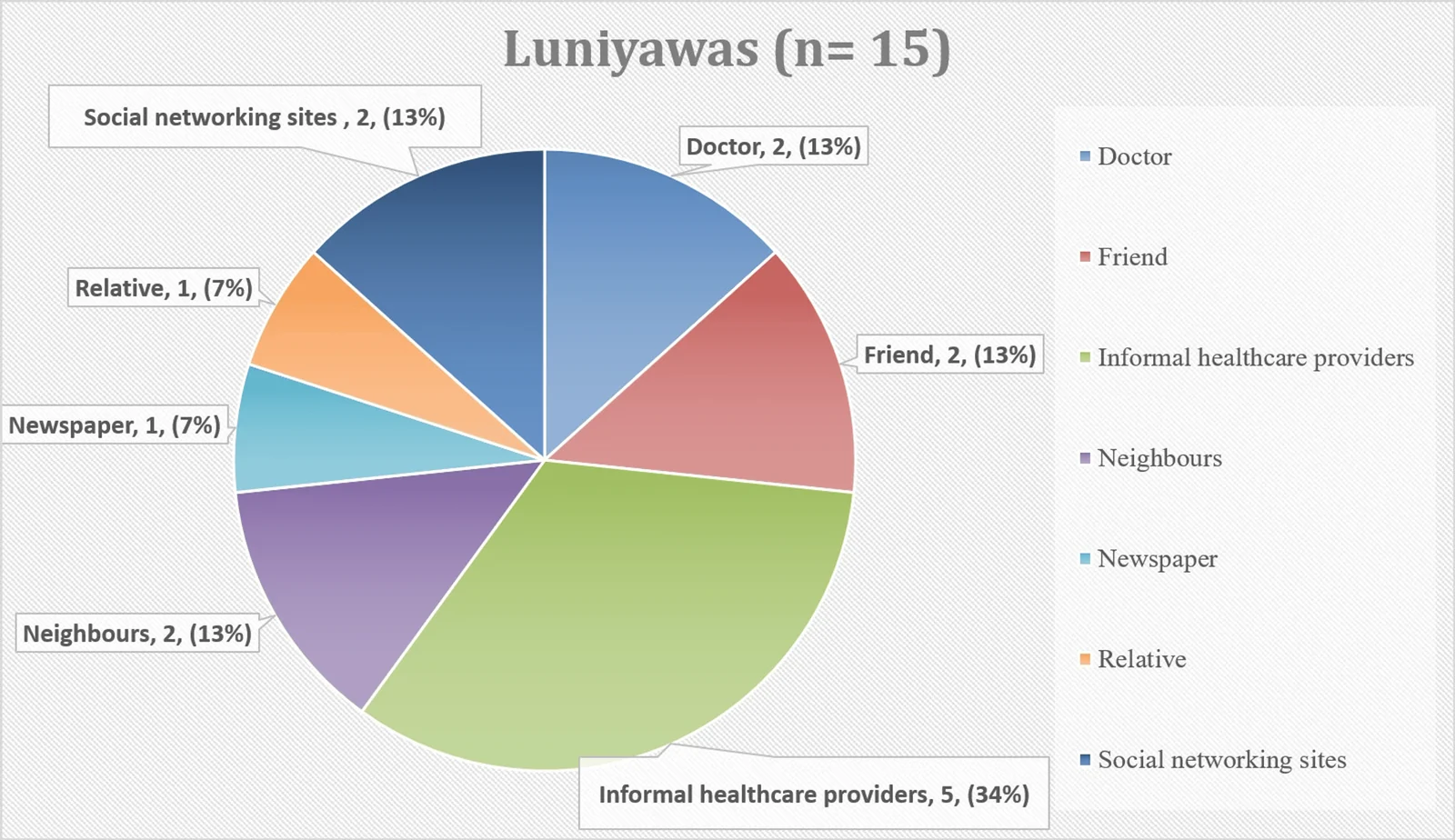
Distribution of study participants according to their sources of health information in Luniyawas (n = 15) Figure created using Microsoft Word (Microsoft® Corp., Redmond, WA).

In Luniyawas (Figure 2), informal healthcare providers were also the major source of health information, reported by five participants (33.3%). Doctors, friends, neighbours and social networking sites were each reported by two participants (13.3%) and contributed equally as sources of health information. Newspapers and relatives were each reported by one participant (6.7%) and constituted the least frequently reported sources. Overall, informal healthcare providers emerged as the predominant source of health information in both study areas.

Table 4 depicts the Quinary prevention strategies employed by the local practitioners in dealing with the spread of diabetes-related misinformation in both the study areas. Counselling of patients was carried out by all practitioners from Khatipura and Luniyawas (five, 100%). Education of family members was reported by half of the practitioners in Khatipura (50%) and nearly two-thirds in Luniyawas (two, 66.7%). Repeated reinforcement was done by all the practitioners from Khatipura (two, 100%); however, only one, i.e., 33.3% of practitioners from Luniyawas, used repeated reinforcement as a method for dealing with the dissemination of misinformation. One (50%) of local practitioners from Khatipura used printed information, education and communication (IEC) material for educating the masses, while only one (33.3%) practitioner from Luniyawas used printed IEC material. Refuting myths related to aetiology and treatment through health awareness camps was reported by one, i.e., 50%, of practitioners from Khatipura and only one, i.e., 33.33%, of practitioners from Luniyawas.

**Table 4:** Quinary prevention strategies employed by local practitioners (N = 5)

Sr. no.	Quinary prevention strategy	Practitioners reporting - Khatipura (n = 2) (n%)	Practitioners reporting - Luniyawas (n = 3) (n%)
1	Counselling of patients	2 (100)	3 (100)
2	Educating the family	1 (50)	2 (66.7)
3	Use of printed IEC material	1 (50)	1 (33.33)
4	Repeated reinforcement	2 (100)	2 (66.7)
5	Refuting myths related to aetiology and treatment through health awareness camps	1 (50)	1 (33.3)

Table 5 shows the barriers faced by local practitioners in countering diabetes-related misinformation in Khatipura (n = 2) and Luniyawas (n = 3). Patient resistance to medical advice and over-reliance on informal healthcare providers were reported by all practitioners in both Khatipura (two, 100%) and Luniyawas (three, 100%). Time constraints due to OPD workload were reported by one practitioner (50%) in Khatipura and all practitioners in Luniyawas (three, 100%). Poor health literacy was identified as a barrier by one practitioner (50%) in Khatipura and two practitioners (66.7%) in Luniyawas. A strong faith in home remedies was reported by one practitioner (50%) in Khatipura and two practitioners (66.7%) in Luniyawas.

**Table 5:** Barriers faced by local practitioners in countering misinformation

Sr. no.	Barriers faced by local practitioners	Practitioners reporting - Khatipura (n = 2) (n%)	Practitioners reporting - Luniyawas (n = 3) (n%)
1	Patient resistance to medical advice	2 (100)	3 (100)
2	Over-reliance on quacks	2 (100)	3 (100)
3	Time constraints (limited time due to OPD load)	1 (50)	3 (100)
4	Poor health literacy	1 (50)	2 (66.7)
5	Strong faith in home remedies	1 (50)	2 (66.7)

## Discussion

This study looked at common misconceptions around the causes and management of T2DM, and how local practitioners are using Quinary prevention to counter them.

Misconceptions about causes

Most participants - 68.75% in Khatipura and 50% in Luniyawas - believed diabetes is caused by eating sugar, and over half in both areas thought it would go away completely if sugar intake stopped. A smaller group (18.75% and 20%) thought repeated blood testing itself causes diabetes, while Babak et al. [15] found 45.3% of their sample believed it was purely hereditary. This mirrors earlier Indian community studies by Rai and Kishore [12] and Nalavadey et al. [14], where diabetes was similarly blamed on sugar intake rather than genetic or metabolic causes - pointing to gaps in basic health literacy.

Misconceptions about medication

Around a third of participants held mistaken beliefs about anti-diabetic drugs, and nearly all of them (100% in Khatipura, 73.3% in Luniyawas) thought allopathic medicines cause organ failure. This tracks with what Shiba et al. [16] and Patil et al. [17] found - fear of drug side effects was the main reason patients skipped medication in favour of diet or home remedies, often at the cost of glycaemic control.

Misconceptions about insulin

Every participant in Khatipura and 80% in Luniyawas saw insulin as a "last resort" treatment, echoing Brod et al. [13] who identified fear and stigma as major barriers to starting insulin therapy. Around half also believed insulin was harmful or habit-forming, similar to Chen et al. [18] in Taiwan, where half their participants feared insulin would damage the kidneys and lead to dialysis.

Misconceptions about monitoring

Nearly all participants (100% Luniyawas, 93.75% Khatipura) felt kidney and lipid tests were unnecessary as long as blood sugar looked normal - a pattern also seen in Shiba et al.'s [16] Haryana-based study. This narrow focus on glucose alone likely contributes to undetected long-term complications.

Misconceptions about cure

Three-quarters of participants believed diabetes could be cured through home remedies, and 68.75% specifically used bitter gourd, coriander, or aloe vera juice - more common in Khatipura than Luniyawas. Nalavadey et al. [14] and Patil et al. [17] reported the same trend, with herbal remedies seen as safer despite no scientific backing.

Physical activity - a bright spot

Unlike most other domains, no misconceptions emerged here: nobody believed exercise was unsafe or that brief yoga sessions could replace sustained aerobic activity. This contrasts with Nalavadey et al. [14], where over half the sample thought diabetics should not exercise at all, and aligns instead with the American Diabetes Association (ADA)/American College of Sports Medicine (ACSM) guidance from Colberg et al. [19] on the benefits of structured activity.

Sources of health information

The pie charts in the present study showed the various sources of health information in both the study areas (Khatipura and Luniyawas). It was observed that in both Khatipura and Luniyawas, informal healthcare providers were the primary source of health information, suggesting greater dependence on non-allopathic practitioners, followed by doctors, friends and neighbours, while social networking sites contributed to a smaller proportion of health-related information. These findings suggest that informal healthcare providers continue to play an important role in influencing diabetes-related beliefs and treatment practices in the study communities. Similar findings have been reported by Almalki et. al., who observed that many individuals with diabetes relied on complementary and alternative sources of treatment and information [20]. This underscores the need for stronger evidence-based health education and communication to improve diabetes management.

Implications for Quinary prevention

These findings underline the importance of local practitioners actively countering misinformation through Quinary prevention - a strategy shown elsewhere [21] to reduce the spread of de-hearsay and improve glycaemic outcomes. Practitioners in this study reported using patient counselling, family education, IEC materials, and repeated reinforcement, consistent with recommended community health education approaches [13]. However, they also cited real barriers: patient resistance, reliance on quacks, heavy OPD loads, poor health literacy, and deep-rooted faith in home remedies - challenges echoed in earlier work on treatment adherence and glycaemic control, as well as systematic reviews highlighting short consultation times, limited provider training, and difficulty supporting behaviour change [22-24].

Limitations

All the study participants were males, and the small sample size may restrict the trends noticed. Secondly, it was conducted in two peri-urban communities of Jaipur; therefore, the findings may not be generalizable to other populations. Lastly, the participants’ responses may have been influenced by recall bias or social desirability bias.

## Conclusions

This qualitative study shows a high burden of diabetes-related misinformation among study subjects residing in Khatipura and Luniyawas. This study showed various misconceptions related to the aetiology, oral anti-diabetic drugs, insulin therapy, investigations and cure of diabetes. The dissemination of misinformation related to diabetes in both study areas was largely attributed to over-reliance on informal practitioners. The efforts of local practitioners in tackling the misinformation involved Quinary prevention measures such as counselling of patients, family awareness, repeated reinforcement, refuting myths related to diabetes and use of IEC materials. However, these efforts were constrained by several factors such as poor health literacy, patient resistance to medical advice, over-reliance on quacks and long-standing faith in home remedies.

The findings of this study highlight the need to formally incorporate Quinary prevention into routine primary care and community-based diabetes programmes. It is crucial to address the widespread dissemination of misinformation in the study population through proper patient education, creating awareness among the family members and spreading reliable health information across social networking sites. Such measures play an important role in countering misinformation related to diabetes and improving diabetes outcomes among the vulnerable and low-literacy populations.

## Appendices

Interview guide for study participants

1. Please introduce yourself briefly (socio-demographic variables - name, age, religion, marital status, type of family, education, occupation, total income, diet preferences, family history, duration of diabetes, alcohol and smoking history).

2. Can you tell us about your journey after being diagnosed with diabetes?

3. What do you know about diabetes?

4. How do you think diabetes usually occurs?

5. Where do you usually obtain information regarding diabetes?

6. What are your views about anti-diabetic drugs, insulin and home remedies for the cure of diabetes?

7. Have you ever heard or received information about diabetes that later turned out to be incorrect?

8. What are some common beliefs or myths about diabetes in your community?

9. How did you decide where to seek treatment after diagnosis?

10. What factors influenced your choice of treatment?

11. Have you ever discussed diabetes-related information that you received from others with your doctor?

12. What difficulties do people face in following correct diabetes treatment?

13. Is there anything else you would like to share regarding diabetes management or misleading information that we have not discussed?

Interview guide for medical practitioners

1. Mutual introduction

2. Experiences in treating diabetes patients in the locality.

3. What are the Quinary prevention strategies employed by you in dealing with patients depending on de-hearsay or quacks for management of T2DM?

4. What are the barriers faced by you in countering misinformation?

5. Is there anything else you would like to share regarding diabetes management or misleading information that we have not discussed?
